# Digitally quantified area of residual tumor after neoadjuvant chemotherapy in HER2-positive breast cancer

**DOI:** 10.1007/s12282-025-01694-7

**Published:** 2025-04-02

**Authors:** Mao Uematsu, Hiromichi Nakajima, Hirohiko Miyake, Masashi Wakabayashi, Chikako Funasaka, Chihiro Kondoh, Kenichi Harano, Nobuaki Matsubara, Ako Hosono, Yoichi Naito, Naoya Sakamoto, Motohiro Kojima, Tatsuya Onishi, Genichiro Ishii, Toru Mukohara

**Affiliations:** 1 Department of Medical Oncology, National Cancer Center Hospital East, 6-5-1 Kashiwanoha, Kashiwa, 277-8577 Japan; 2 Course of Advanced Clinical Research of Cancer, Juntendo University Graduate School of Medicine, Bunkyō, Japan; 3 Department of Experimental Therapeutics, National Cancer Center Hospital East, 6-5-1 Kashiwanoha, Kashiwa, 277-8577 Japan; 4 Department of General Internal Medicine, National Cancer Center Hospital East, 6-5-1 Kashiwanoha, Kashiwa, 277-8577 Japan; 5 Department of Pathology and Clinical Laboratories, National Cancer Center Hospital East, 6-5-1 Kashiwanoha, Kashiwa, 277-8577 Japan; 6 Biostatistics Division, Center for Research Administration and Support, National Cancer Center, Chuo-Ku, Japan; 7 Department of Pediatric Oncology, National Cancer Center Hospital East, 6-5-1 Kashiwanoha, Kashiwa, 277-8577 Japan; 8 Exploratory Oncology Research and Clinical Trial Center, Division of Pathology, Chuo-Ku, Japan; 9 Department of Breast Surgery, National Cancer Center Hospital East, 6-5-1 Kashiwanoha, Kashiwa, 277-8577 Japan

**Keywords:** HER2-positive breast cancer, Area of residual tumor, Neoadjuvant chemotherapy, Biomarker, Digital pathology

## Abstract

**Background:**

The area of residual tumor (ART) is a quantitative method for assessing tumors after neoadjuvant chemotherapy (NAC). This study evaluated whether ART can identify a favorable prognosis group in patients with HER2-positive surgically resected breast cancer and residual tumors post-NAC.

**Methods:**

We retrospectively reviewed patients with HER2-positive who underwent surgery after NAC, including trastuzumab, from 2005 to 2022 at our institution. ART was assessed at the maximum cut surface of the residual primary tumor using digital pathology images. Receiver operating characteristic curve analysis determined ART-Low and ART-High cutoffs, excluding ART-0 (0 mm^2^) patients.

**Results:**

Of the 219 patients, 82 had ART greater than 0 mm^2^. The median follow-up was 90.2 months. The number of patients in the ART-0, ART-Low (0 < ART ≤ 4.0 mm^2^), and ART-High (> 4.0 mm^2^) groups were 137, 39, and 43, respectively. The ART-Low group showed significantly shorter event-free survival compared to the ART-0 group (HR 3.50, 95% CI 1.52–8.06), and the ART-High group also tended toward poorer prognosis (HR 2.31, 95% CI 0.89–5.97). However, there was no significant difference in prognosis between the ART-Low and ART-High groups.

**Conclusions:**

The current study suggests that even minimal residual tumor cells in the primary site can significantly impact on prognosis in HER2-positive early breast cancer.

**Supplementary Information:**

The online version contains supplementary material available at 10.1007/s12282-025-01694-7.

## Introduction

The standard treatment for human epidermal growth factor receptor 2 (HER2)-positive early breast cancer involves a combination of surgery, chemotherapy with anti-HER2 therapy, and radiation therapy. Historically, chemotherapy was administered as either neoadjuvant or adjuvant chemotherapy. However, there has been a shift toward a residual disease-guided approach, positioning neoadjuvant chemotherapy (NAC) as the preferred option. The KATHERINE study aimed to improve the prognosis of high-risk patients with HER2-positive breast cancer. This trial targeted high-risk patients—specifically, those without a pathological complete response (non-pCR), defined as ypT0/Tis and ypN0, after receiving NAC with trastuzumab. Adjuvant trastuzumab emtansine (T-DM1) demonstrated better outcomes compared to adjuvant trastuzumab alone [1]. As a result, the residual disease-guided approach has become the standard treatment: trastuzumab (± pertuzumab) is used in patients with pCR after NAC, while the more intensive T-DM1 is administered to patients with non-pCR [2]. However, T-DM1 caused increased incidences of thrombocytopenia and chemotherapy-induced peripheral neuropathy, as well as a potential decline in quality of life compared to trastuzumab. Therefore, it is crucial to refine the truly poor prognosis population who need intensified treatment by adjuvant T-DM1 therapy among patients who resulted in non-pCR after NAC.

In patients with early-stage breast cancer, pCR after NAC is associated with favorable long-term outcomes [3], and particularly in HER2-positive breast cancer, pCR is strongly prognostic for event-free survival (EFS) and overall survival (OS) [4]. pCR is generally defined as the absence of invasive cancer in the resected breast specimen and all sampled ipsilateral lymph nodes (ypT0/Tis, ypN0). Several measures, including the ypTNM classification, residual cancer burden (RCB), and Neo-Bioscore, are used to evaluate prognosis after NAC. RCB combines tumor bed size, cellularity, lymph node involvement, and other factors to classify patients into four categories (RCB 0–III) [5, 6], which is useful for predicting prognosis. However, it can be complex to assess. Neo-Bioscore refines prognosis by integrating clinical and pathological stages, estrogen receptor (ER)/HER2 status, and nuclear grade, stratifying patients into eight categories [7]. However, challenges remain in measuring the size of residual tumors (ypT), particularly in cases where multiple scattered invasive foci are found within fibrotic tissue.

We have reported a novel objective and quantitative pathological evaluation method, called the area of residual tumor (ART), to assess residual tumors after NAC. Unlike other methods, ART measurement focuses on direct tumor area quantification, eliminating the need to estimate tumor bed dimensions or calculate residual-to-bed ratios. ART stratified prognosis in various types of cancers [8–13]. Through digital pathology images, we divided 143 patients with triple-negative breast cancer (TNBC) following NAC into three categories: ART-0 (0 mm^2^), ART-Low (0–136 mm^2^), and ART-High (> 136 mm^2^) [12]. We reported that ART-0 and ART-Low exhibited better recurrence-free survival or OS compared to ART-High; however, there was no significant difference between the ART-0 and ART-Low groups. We concluded that the ART-Low group had a favorable outcome in patients with non-pCR and TNBC, suggesting a potential to omit adjuvant capecitabine [14].

We hypothesized that ART could stratify prognosis in patients with non-pCR and HER2-positive early breast cancer after NAC. The objective of this study was to identify a subset of patients with non-pCR and a favorable prognosis. If this study could help identify a subgroup of patients with non-pCR and a prognosis similar to that of pCR, it may allow for avoiding unnecessary adverse events and declines in quality of life associated with T-DM1.

## Material and methods

### Patients

We retrospectively reviewed the medical records of patients with HER2-positive early breast cancer who underwent surgery after NAC, including trastuzumab, at the National Cancer Center Hospital East between April 2005 and May 2022. The study excluded patients involving bilateral breast cancer, synchronous bilateral breast cancer, and metastatic breast cancer (Figure S1).

The study utilized specimens under comprehensive consent from the National Cancer Center, adhering to established guidelines. Protocol approval was obtained from the Institutional Ethical Review Committee (No. 2023–295).

### Pathological analysis

Surgical specimens underwent fixation in 10% formalin solution. Partial mastectomy tissue processing involved sectioning at 5–10 mm intervals. Complete mastectomy specimens were cut into similar thickness slices, focusing on tumor regions, adjacent tissue, and nipple-mass interfaces. Each sample underwent paraffin embedding followed by preparation of 4 µm sections. Hematoxylin and eosin (H&E)-stained slides of the largest residual tumor slice were scanned using the NanoZoomer Digital Pathology Virtual Slide Viewer (Hamamatsu Photonics KK, Hamamatsu, Japan). The areas with residual tumor cells were marked for further analysis.

### Pathological evaluation of neoadjuvant chemotherapy

#### ART

**Fig. 1:**
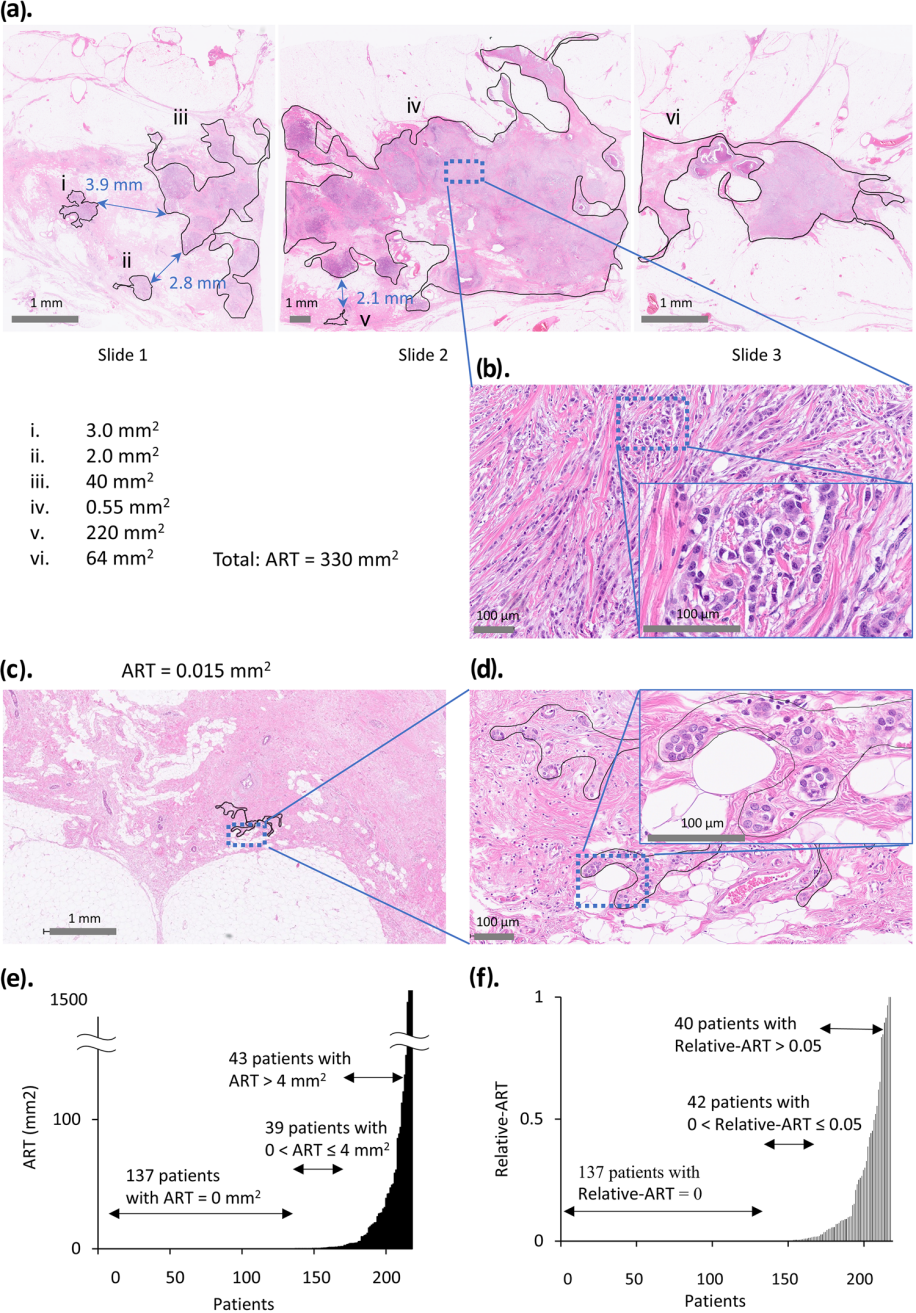
**a** Measurement of the area of residual tumor in a patient with abundant cancer cells remaining after neoadjuvant chemotherapy (NAC) using mastectomy specimens. These three slides show six lesions, each more than 2 mm apart, with a total area of 332.6 mm^2^. **b** Higher magnification image of the square in (**a**). **c** Patient with only a small number of cancer cells remaining after NAC. **d** Higher magnification image of the square in (**c**). **e** Histogram of the area of residual tumor (ART) for each patient. **f** Histogram of relative-ART for each patient. ART, area of residual tumor

We evaluated ART by examining the maximum tumor section from each surgical specimen, following previous protocols (Table S1) [8–10]. The evaluation process employed the NanoZoomer Digital Pathology Virtual Slide Viewer. Our assessment incorporated cancer cells showing degeneration while maintaining cellular components. The analysis excluded any necrotic regions along with intraepithelial components [8, 12]. Following established reports, we considered tumor cell clusters as independent units when the distance between them exceeded 2 mm [12]. The measurement of ART was first conducted by the medical oncologist M.U and then confirmed by the pathologist H.M. ART was calculated as the sum of all tumor areas in the section showing the largest tumor size. Figure 1a–d represents images of the actual measurements. Slides 1 through 3 showed the maximum cut surfaces of a patient’s residual tumor (Fig. 1a). On slide 1, three lesions, each spaced more than 2 mm apart, were assessed for their respective areas; similarly, two lesions were evaluated on slide 2, and one lesion on slide 3. The total area from these three slides amounted to an ART of 332.6 mm^2^.

#### Relative-ART

We estimated the tumor bed by considering the tumor size on pre-NAC imaging and the extent of fibrosis and granulation tissue. Relative-ART, defined as the ratio of the area of residual tumor to the entire tumor bed area, was calculated to provide a quantitative assessment of the proportion of remaining tumor after neoadjuvant chemotherapy (Table S1).

#### RCB

The RCB classifies post-NAC tumor burden into four distinct levels (Table S1). RCB 0 represents pCR (ypT0/Tis, N0) in both breast and lymph nodes, while RCB I signifies minimal disease presence. Moderate residual tumor corresponds to RCB II, and extensive disease is designated as RCB III [5, 15]. M.U. evaluated surgical specimens (breast and nodal tissue) from non-pCR patients to determine RCB values using established protocols. Any challenging cases requiring clarification were jointly assessed with pathologist H.M.

### Statistical analysis

We defined EFS as the duration between surgical intervention and either distant disease progression or breast cancer mortality. Data from patients who remained metastasis-free at final observation or died from alternative causes underwent censoring at respective time points. Analysis of survival differences utilized Kaplan–Meier method and Log-rank test. Hazard ratios (HRs) and their 95% confidence intervals (CIs) were estimated by Cox proportional hazards model incorporating ART alongside established prognostic variables, including age, clinical T stage, clinical N stage, ER status, and histological grade.

Receiver operating characteristic (ROC) curve analysis was performed to determine optimal thresholds for predicting disease recurrence. The study identified ART cutoff values through evaluation of minimal distances to ROC curve peaks, excluding cases where ART measured 0 mm^2^.

Group comparisons employed Fisher's exact test for categorical data, while continuous variables underwent Wilcoxon rank analysis. Data collection continued through August 1, 2024. Statistical analyses utilized EZR software (Saitama Medical Center, Jichi Medical University, Saitama, Japan), which provides R-based analysis tools (The R Foundation for Statistical Computing, Vienna, Austria) [16]. Statistical significance was set at *P* < 0.05.

## Results

### Clinicopathological features

We identified 219 patients from a cohort of 236 who received trastuzumab-containing NAC (Figure S1). Patient characteristics appear in Table 1. NAC regimens comprised anthracycline–taxane combination in 175 patients (79.9%), while 44 patients (20.1%) received taxane-based therapy. All patients received NAC with trastuzumab: 166 (75.8%) received trastuzumab alone, and 53 (24.2%) received a combination of trastuzumab and pertuzumab. Adjuvant chemotherapy was administered to 96.3% of the patients. Among them, 163 (74.4%) were treated with trastuzumab monotherapy, 39 (17.8%) with trastuzumab and pertuzumab, and 7 (3.2%) with T-DM1.

**Table 1:** Patient and treatment characteristics

Characteristics		*N* (%)
Clinical factors		
Age	Median (range)	55 (31–80)
Clinical T stage	0	2 (0.9)
	1	18 (8.3)
	2	140 (63.9)
	3	38 (17.4)
	4	21 (9.6)
Clinical N stage	Negative	66 (30.1)
	Positive	153 (69.9)
Clinical stage	1	9 (4.2)
	2	128 (58.4)
	3	82 (37.4)
Pathological factors		
Histology	Invasive ductal carcinoma	208 (95.4)
	Invasive lobular carcinoma	2 (0.9)
	Other	8 (3.7)
Histological grade^a^	1–2	189 (86.3)
	3	16 (7.3)
	Unknown	14 (6.4)
Hormone receptor status^a^	ER status positive	96 (43.8)
	PgR status positive	60 (27.6)
Ki-67^a^	> 20%	140 (70.7)
Neoadjuvant chemotherapy		
Cytotoxic agents	Anthracycline/taxane	175 (79.9)
	Taxane based	44 (20.1)
	PTX	35 (16.0)
	DTX/PTX + CBDCA	6 (2.7)
	Other	3 (1.4)
Anti-HER2 agents	Trastuzumab only	166 (75.8)
	Trastuzumab and pertuzumab	53(24.2)
Surgery		
Breast	Breast-conserving surgery	106 (48.4)
	Mastectomy	112 (51.1)
	No surgical resection of the primary tumor^b^	1 (0.5)
Lymph node	Sentinel lymph node biopsy	61 (27.9)
	Axillary lymph node dissection	118 (53.9)
Adjuvant chemotherapy		
Adjuvant treatment	Yes	211 (96.3)
Anti-HER2 agents	Trastuzumab only	163 (74.4)
	Trastuzumab and pertuzumab	39 (17.8)
	T-DM1	7 (3.2)
	Investigational treatments	2 (1.0)
Adjuvant endocrine therapy	Yes	86 (39.3)

^a^Histological grade, hormone receptor status, and Ki67 index were assessed from pre-treatment biopsy specimens

^b^This case was occult breast cancer with lymph node metastasis and underwent lymph node dissection only, resulting in no measurable residual tumor and was classified as area of residual tumor (ART)−0

*CBDCA* carboplatin, *DTX* docetaxel, *ER* estrogen receptor, *HER2 *human epidermal growth factor receptor 2, *PgR* progesterone receptor, *PTX* paclitaxel, *T-DM1* trastuzumab emtansine

### Evaluation of ART

Figure 1a represents a section of the largest tumor slice from a case where numerous cancer cells remained after NAC, with Fig. 1b offering a magnified view of the same area. The ART, in this case, was 330 mm^2^. Figure 1c shows a patient with minimal residual cancer cells, while Fig. 1d provides a closer view of Fig. 1c. In this case, the ART was 0.015 mm^2^. Overall, 137 patients had no primary residual tumor, with an ART of 0 mm^2^ (ART-0), and the median ART of patients excluded ART-0 was 4.2 mm^2^ (range, 0.0012–1700 mm^2^) (Fig. 1e). The median relative-ART of patients excluded relative-ART was 0.050 (range, 0.00012–1.0) (Fig. 1f).

### Clinicopathological features stratified by ART status

**Fig. 2:**
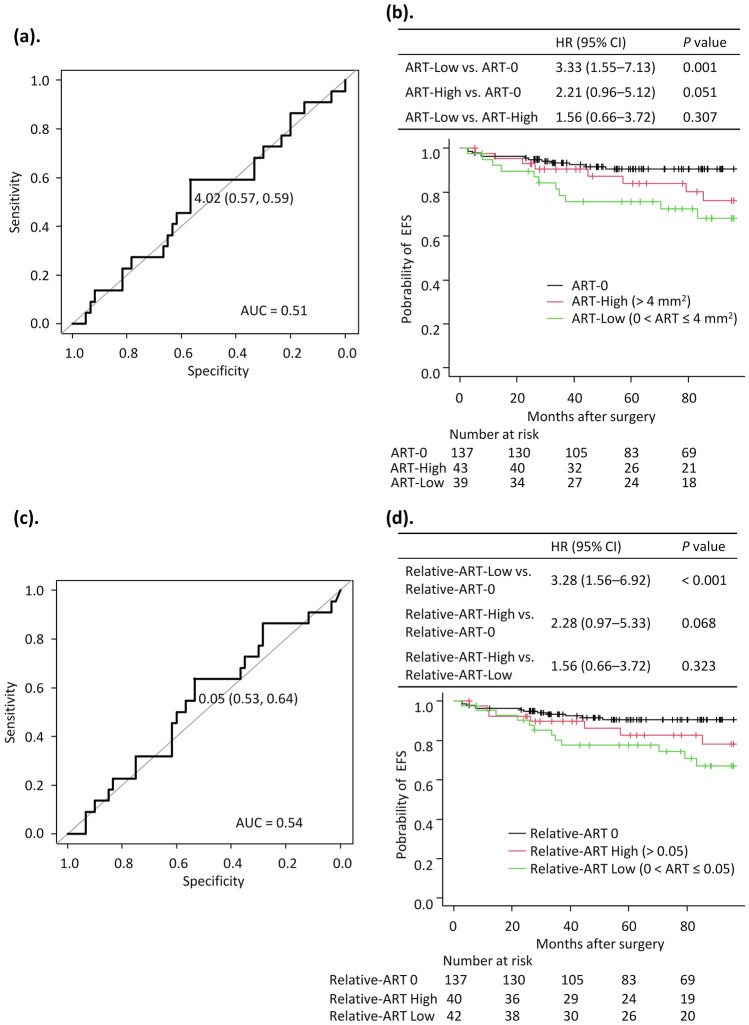
**a** Receiver operating characteristic (ROC) curve for determining the area of residual tumor (ART) threshold in HER2-positive breast cancer. The area under the ROC curve (AUC) was 0.513, and the cutoff was set at 4.0 mm^2^. **b** Kaplan–Meier survival curves for event-free survival (EFS) in patients with HER2-positive breast cancer, stratified by ART groups: ART-0, ART-Low, and ART-High. **c** ROC curve for determining the relative ART threshold in HER2-positive breast cancer. The AUC was 0.54, and the cutoff was set at 0.05. **d** Kaplan–Meier survival curves for EFS in patients with HER2-positive breast cancer, stratified by relative-ART groups: relative-ART 0, relative-ART Low, and relative-ART High. *AUC* area under the curve, *ART* area of residual tumor, *EFS* event-free survival, *CI* confidence interval, *HR* hazard ratio

The ROC curve for ART against EFS was created after excluding the 137 patients with ART-0 and revealed an area under the curve (AUC) of 0.51 (Fig. 2a). The cutoff value for ART was set at 4.0 mm^2^, determined using the ROC curve by minimizing the distance to ROC curve peaks. The median ART value of 4.2 mm^2^ in non-ART-0 patients further supported this cutoff. Based on this cutoff, patients were classified into two groups: ART-Low (0 < ART ≤ 4.0 mm^2^), consisting of 39 patients, and ART-High (> 4.0 mm^2^), consisting of 43 patients.

Table 2 presents correlations between ART status and patient characteristics. The ART-Low population exhibited higher clinical T stage, ER and PgR positivity, higher ypT and ypN stage, and less frequent use of neoadjuvant pertuzumab and trastuzumab than the ART-0 group. Compared to ART-Low, the ART-High group exhibited more lymphovascular invasion with higher ypT and numerically high ypN. Similarly, when comparing the ART-High group to ART-0, significant differences were observed in ER and PgR positivity, ypT and ypN stages, lymphovascular invasion, and HER2 IHC score 3 + before NAC.

**Table 2:** Comparison of clinicopathological features according to area of residual tumor (ART) status

Characteristics	ART-0 *N* = 137	ART-Low *N* = 39	ART-High *N* = 43	*P* value		
				ART-Low vs. ART-0	ART-High vs. ART-0	ART-Low vs ART-High
Age—median (Range)	55 (31–76)	54 (36–73)	54 (37–80)	0.641	0.266	0.575
Clinical T stage						
T0/1/2	107 (78.1)	23 (59.0)	30 (69.8)	0.023	0.306	0.359
T3/4	30 (21.9)	16 (41.0)	13 (30.2)			
Clinical N stage						
Negative	42 (30.7)	11 (28.2)	13 (30.2)	0.845	1.000	1.000
Positive	95 (69.3)	28 (71.8)	30 (69.8)			
Clinical stage						
CStage 1/2	88 (64.2)	21 (53.8)	28 (65.1)	0.265	1.000	0.369
cStage 3	49 (35.8)	18 (46.2)	15 (34.9)			
ER status^a^						
Negative	95 (69.3)	14 (35.9)	14 (32.6)	< 0.001	< 0.001	0.818
Positive	42 (30.7)	25 (64.1)	29 (67.4)			
PgR status^a^						
Negative	111 (81.0)	20 (51.3)	26 (63.4)	< 0.001	0.033	0.366
Positive	26 (19.0)	19 (48.7)	15 (36.6)			
HER2^a^						
2 + , FISH positive	6 (4.4)	3 (7.7)	6 (14.0)	0.417	0.039	0.487
HER2 3 +	131 (95.6)	36 (92.3)	37 (86.0)			
Ki-67^a^—median (range)	30 (1–80)	30 (1–80)	30 (5–70)	0.942	0.032	0.071
Histological grade^a^						
1–2	117 (85.4)	32 (82.1)	40 (93.0)	0.743	0.297	0.179
3	11 (8.0)	4 (10.3)	1 (2.3)			
Unknown	9 (6.6)	3 (7.7)	2 (4.7)			
ypT stage						
T0/1	137 (100.0)	36 (92.3)	28 (65.1)	0.010	< 0.001	0.003
T2/3/4	0 (0.0)	3 (7.7)	15 (34.9)			
ypN stage						
Negative	129 (94.2)	32 (82.1)	28 (65.1)	0.025	< 0.001	0.133
Positive	8 (5.8)	7 (17.9)	15 (34.9)			
Lymphovascular invasion						
No	136 (99.3)	38 (97.4)	33 (76.7)	0.395	< 0.001	0.008
Yes	1 (0.7)	1 (2.6)	10 (23.3)			
Neoadjuvant pertuzumab	40 (29.2)	4 (10.3)	9 (20.9)	0.020	0.331	0.234

^a^ER, PgR, HER2 status, Ki67 index, and histological grade were assessed from pre-treatment biopsy specimens

*ART* antiretroviral therapy, *ER* estrogen receptor, *FISH* fluorescence in situ hybridization, *HER2* human epidermal growth factor receptor 2, *PgR* progesterone receptor, *ypT* post-therapy primary tumor stage

### Prognostic role of ART and relative-ART

The median follow-up period was 90.2 months (range: 81.6–97.2). 3-year EFS was 93.3% (95% CI 87.4–96.4) for ART-0, 78.5% (95% CI 61.5–88.6) for ART-Low, and 90.3% (95% CI 76.3–96.3) for ART-High. Analysis revealed inferior EFS in ART-Low versus ART-0 patients (HR 3.33, 95% CI 1.55–7.13, *P* = 0.001). The ART-High group tended to exhibit shorter EFS compared with ART-0 (HR 2.21, 95% CI 0.96–5.12, *P* = 0.051). EFS comparison between ART-High and ART-Low groups demonstrated no significant differences (HR 1.56, 95% CI 0.66–3.72, *P* = 0.307) (Fig. 2b).

A subgroup analysis based on hormone receptor status was conducted. In patients with hormone receptor-positive, ART-0 had a 3-year EFS of 93.4%. In comparison, ART-Low showed a significantly lower 3-year EFS of 78.7% (HR 6.01, 95% CI 1.62–22.28, *P* = 0.002), while ART-High had a 3-year EFS of 92.9% (HR 2.64, 95% CI 0.63–11.08, *P* = 0.184) (Figure S2a). No significant difference was observed in EFS between the ART-High and ART-Low populations. In the patients with hormone receptor-negative subgroup, the 3-year EFS reached 93.2% (95% CI 85.5–96.9) for ART-0, 83.9% (95% CI 57.9–94.5) for ART-Low, and 77.8% (95% CI 36.5–93.9) for ART-High (Figure S2b). Survival comparison showed comparable EFS between ART-0 and ART-Low groups (HR 2.09, 95% CI 0.57–7.61, *P* = 0.252). ART-High patients demonstrated a tendency toward shorter EFS versus ART-0 (HR 2.70, 95% CI 0.85–8.62, *P* = 0.081). EFS remained similar between ART-High and ART-Low (HR 0.76, 95% CI 0.17–3.40, *P* = 0.718).

In addition, a subgroup analysis by adjuvant treatment showed that in patients receiving hormonal therapy, the ART-Low group had significantly shorter EFS compared to the ART-0 group (HR 4.31, 95% CI 1.12–16.53, *P* = 0.021). In patients without hormonal therapy, both the ART-Low and ART-High groups tended to have shorter EFS than the ART-0 group (HR 2.91, 95% CI 1.01–8.40, *P* = 0.039, and HR 2.53, 95% CI 0.88–7.29, *P* = 0.075). In patients receiving trastuzumab monotherapy, both the ART-Low and ART-High groups showed significantly poorer prognosis compared to the ART-0 group (HR 3.68, 95% CI 1.51–8.99, *P* = 0.002, and HR 3.69, 95% CI 1.42–9.57, *P* = 0.004). Patients who received trastuzumab and pertuzumab included only two ART-High patients and several ART-0. In contrast, patients treated with T-DM1 comprised five ART-High and two ART-Low patients, with no ART-0. These sample size differences make meaningful statistical comparisons between these treatment groups challenging. There were no significant differences in prognosis between ART-Low and ART-High across adjuvant treatment subgroups.

The ROC curve for relative-ART in relation to EFS was plotted after excluding the 137 patients with a relative-ART of 0 (relative-ART 0), resulting in an AUC of 0.54 (Fig. 2c). A cutoff value of 0.05 was established, defining the groups as relative-ART Low (0 < ART ≤ 0.05) with 42 patients and relative-ART High (> 0.05) with 40 patients. The relative-ART Low group demonstrated significantly poorer EFS compared to the relative-ART 0 group (HR 3.28, 95% CI 1.56–6.92, *P* < 0.001). The relative-ART High group showed a trend toward shorter EFS compared to relative-ART 0 (HR 2.28, 95% CI 0.97–5.33, *P* = 0.068). However, relative-ART analysis showed no significant EFS difference EFS between High and Low groups (HR 1.56, 95% CI 0.66–3.72, *P* = 0.323) (Fig. 2d).

Univariable analysis identified four significant EFS predictors: ART, clinical T stage, disease stage, and ypN status. Multivariable analysis demonstrated significantly shorter EFS for ART-Low vs. ART-0 (HR 3.50, 95% CI 1.52–8.06) and a trend for shorter EFS for ART-High vs. ART-0 (HR 2.31, 95% CI 0.89–5.97) (Table 3).

**Table 3:** Univariable and multivariable analyses of clinicopathological factors for event-free survival

Variable	*n*	Univariable analysis			Multivariable analysis		
		Hazard ratio	95% CI	*P* value	Hazard ratio	95% CI	*P* value
Age (year)							
< 40	19	Ref			Ref		
≥ 40	200	0.42	0.18–1.02	0.056	0.44	0.17–1.14	0.090
Clinical T stage							
T0/1/2	160	Ref			Ref		
T3/4	59	3.46	1.79–6.69	< 0.001	2.32	1.07–5.05	0.034
Clinical N stage							
Negative	66	Ref			Ref		
Positive	153	1.43	0.67–3.04	0.357	1.19	0.50–2.81	0.697
Clinical stage							
cStage 1/2	137	Ref					
cStage 3	82	4.32	2.12–8.78	< 0.001			
ER status^a^							
Negative	123	Ref			Ref		
Positive	96	1.06	0.55–2.05	0.855	0.73	0.35–1.53	0.405
Ki-67^a^							
≤ 20	79	Ref					
> 20	140	1.46	0.65–3.29	0.358			
Histological grade^a^							
1–2	189	Ref			Ref		
3	16	1.57	0.55–4.48	0.399	1.12	0.38–3.32	0.843
ypN stage							
Negative	189	Ref					
Positive	30	2.73	1.31–5.71	0.008			
Lymphovascular invasion							
No	207	Ref					
Yes	12	1.81	0.55–5.93	0.326			
ART							
ART-0	137	Ref			Ref		
ART-Low	39	3.33	1.55–7.13	0.002	3.50	1.52–8.06	0.003
ART-High	43	2.21	0.96–5.12	0.064	2.31	0.89–5.97	0.084

^a^ER status, Ki67 index, and histological grade were assessed from pre-treatment biopsy specimens

*ART *anti-retroviral therapy, *CI* confidence interval, *ER* estrogen receptor, *HR* hazard ratio, *ypN* pathological N stage after neoadjuvant therapy

### Relation between ART and RCB

**Fig. 3:**
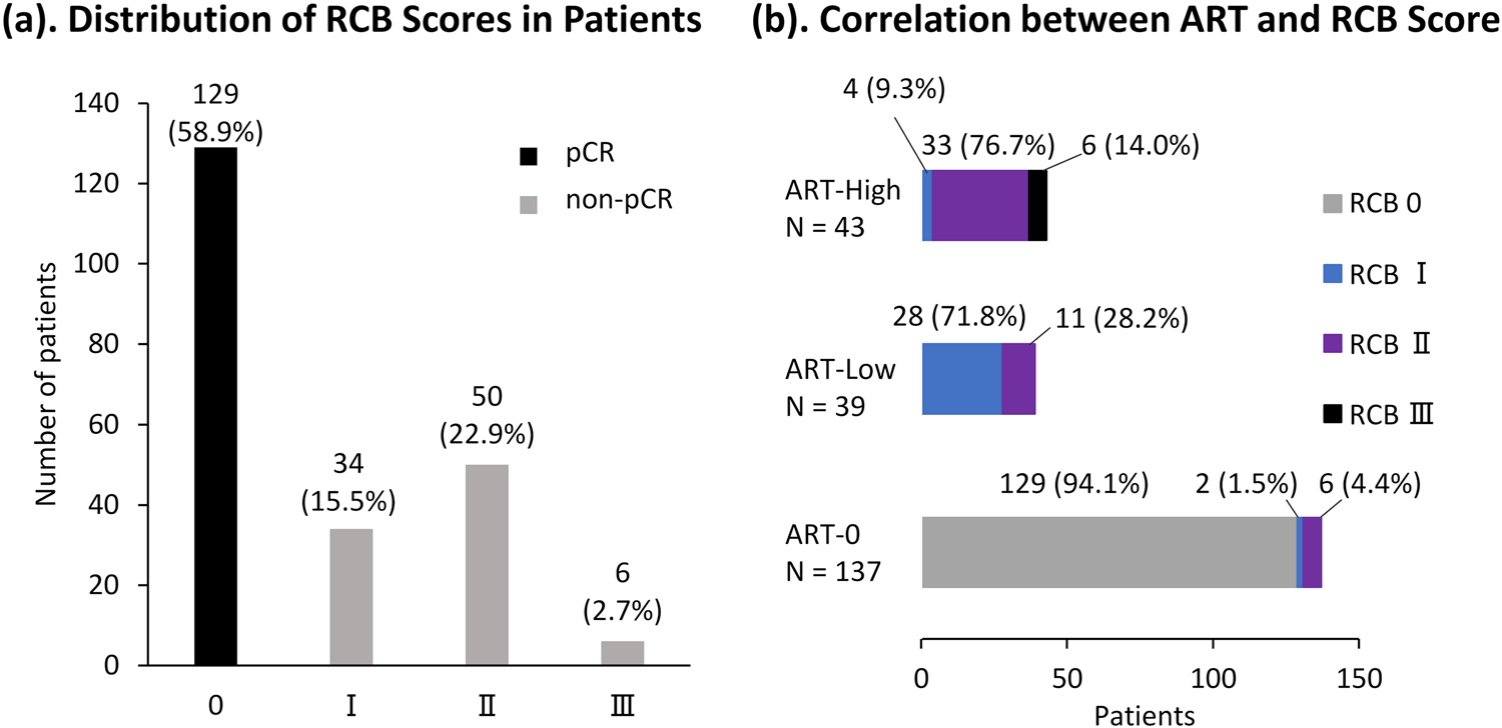
**a** Distribution of residual cancer burden (RCB) scores. Black = pathological complete response (pCR), light gray = residual disease. **b** Relationship between the area of residual tumor (ART) and RCB score, illustrating how ART correlates with varying degrees of residual disease based on RCB classification. *ART* area of residual tumor, *RCB* residual cancer burden

RCB analysis categorized 129 patients (58.9%) as RCB 0. Among patients showing residual disease, classifications included 34 patients (15.5%) as RCB I, 50 (22.9%) as RCB II, and 6 (2.7%) as RCB III (Fig. 3a). Among the ART-0 group, 129 patients (94.1%) had RCB 0, 2 patients (1.5%) had RCB I, and 6 patients (4.4%) had RCB II. In the ART-Low group, 28 patients (71.8%) had RCB I, while 11 patients (28.2%) had RCB II. The ART-High group comprised 4 patients (9.3%) with RCB I, 33 patients (76.7%) with RCB II, and 6 patients (14.0%) with RCB III (Fig. 3b). The 3-year EFS for RCB 0, RCB I, RCB II, and RCB III were 93.6% (95% CI 87.6–96.8), 87.8% (95% CI 70.7–95.3), 83.2% (95% CI 69.2–91.2), and 83.3% (95% CI 27.3–97.5), respectively (Figure S4).

## Discussion

This is the first study to investigate whether ART can stratify prognosis among patients with non-pCR in HER2-positive breast cancer. No significant difference in survival was observed between the ART-Low and ART-High groups. The findings revealed that ART was not an independent prognostic factor for residual tumors after NAC in HER2-positive breast cancer, in contrast to its effectiveness in TNBC.

We have previously reported that ART could predict outcomes in various types of cancers [8–13]. In TNBC, ART successfully stratified the patients with non-pCR, with ART-Low showing a prognosis comparable to that of ART-0 [12]. However, we could not reproduce these results in HER2-positive breast cancer. A few potential reasons could explain this difference. First, patients with non-pCR in HER2-positive breast cancer who have undergone NAC, including anti-HER2 therapy, represent a heterogeneous group. The loss of HER2 expressions or intratumoral heterogeneity in HER2 expression was reported to affect prognosis [17–19]. *PIK3CA* mutations are known to drive resistance to anti-HER2 therapies, and these mutations are associated with a reduced pCR rate and lower treatment efficacy [20]. Furthermore, predictive analyses of residual tumors through gene expression profiling have shown that patients with luminal subtypes were less sensitive to chemotherapy but had a better prognosis than those with basal-like profiles [21]. In this study, hormone receptor status revealed different trends: hormone receptor-positive patients demonstrated inferior survival in ART-Low versus ART-0 groups, whereas in the hormone receptor-negative group, the ART-High group showed poorer prognosis compared to the ART-0 group. These findings were supported by analyses stratified by adjuvant hormonal therapy, which showed similar prognostic implications of ART status (Figure S3a, S3b). These suggest that the non-pCR group in HER2-positive breast cancer is highly diverse in terms of drug sensitivity and recurrence risk and that the relationship between residual tumor volume and recurrence risk may not be as straightforward as it is in TNBC.

Secondly, the types of drugs used in NAC differ between TNBC and HER2-positive breast cancer. The traditional NAC treatments for TNBC mainly used cytotoxic agents [22], while immune checkpoint inhibitors have been added recently. The effect of cytotoxic agents targets rapidly proliferating cells during specific cell cycle phases [23]. This selective pressure by a single modality often results in the survival of chemo-resistant cells [24], which results in the correlation between the burden of residual tumors and prognosis in TNBC. In contrast, HER2-positive breast cancer combines these chemotherapies with anti-HER2 therapy, such as trastuzumab and pertuzumab [25–28]. The anti-HER2 therapy effectively targets HER2 overexpression by inhibiting HER2-mediated growth signals and promoting antibody-dependent cellular cytotoxicity, regardless of their cell cycle phase [29, 30]. Consequently, NAC including anti-HER2 therapy shows a higher pCR rate compared to TNBC. However, the residual tumor cells exhibit various resistant mechanisms to cytotoxic agents and anti-HER2 therapy [29, 30]. Even small quantities of these residual cells can significantly impact recurrence, which may explain why ART did not correlate with prognosis in HER2-positive breast cancer. In this study, the median ART for HER2-positive breast cancer was 4.2 mm^2^, while for TNBC, it was 29.1 mm^2^ [12]. Our research revealed that the prognosis of ART-Low was clearly worse compared with ART-0 in HER2-positive breast cancer. This indicates that the differences in the modality of NAC regimens between TNBC and HER2-positive breast cancer may be related to the differing prognostic implications of ART. Moreover, our subgroup analyses demonstrated that the prognostic value of ART varied depending on the adjuvant treatment received (Figure S3). These findings suggest that the interaction between NAC regimens and subsequent treatments further complicates the relationship between residual tumor burden and prognosis.

While ART evaluated the remaining tumor based on absolute area, RCB comprehensively assessed multiple factors, including the diameter of the tumor bed, lymph node metastasis, and cellular density. Our study showed higher RCB I frequency in ART-Low group, whereas RCB II and RCB III predominated in the ART-High. This indicated that ART showed a distribution similar to that of RCB. Large-scale studies have shown that RCB effectively predicts prognosis in HER2-positive breast cancer [15, 31], and our study confirmed its prognostic value (Figure S4). However, ART was not successful in predicting prognosis in patients with non-pCR. Additionally, relative-ART, which evaluated the proportion of invasive cancer in the estimated tumor bed, failed to predict prognosis. Because RCB considers multiple factors including lymph node metastases, it provides more comprehensive information about the tumor's characteristics and the risk of metastasis [5]. Therefore, RCB may be more effective than ART in prognostic stratification for HER2-positive breast cancer. This suggests that in HER2-positive breast cancer, both primary lesion and lymph node metastases may be important factors influencing prognosis.

This study has several limitations. Our single-institution retrospective analysis included modest sample size, with particularly limited events in HER2-positive versus TNBC populations. Secondly, there was heterogeneity in treatment regimens both before and after surgery. Additionally, few patients received the current standard adjuvant treatment, T-DM1. Furthermore, our evaluations did not assess the biological heterogeneity in patients with non-pCR beyond HER2 and hormone receptor status.

In conclusion, ART could not identify a favorable prognosis in patients with residual tumors after NAC. These findings suggest that even patients with minimal residual tumors had worse outcomes compared to those with no residual tumor in HER2-positive early breast cancer unlike TNBC, and therefore, adjuvant T-DM1 should not be omitted.

## Supplementary Information

Below is the link to the electronic supplementary material.

Supplementary file1 (TIF 35 KB)

Supplementary file2 (TIF 60 KB)

Supplementary file3 (TIF 864 KB)

Supplementary file4 (TIF 383 KB)

Supplementary file5 (TIF 43 KB)

## Data Availability

The research data supporting this study are restricted for privacy protection of patient information. However, anonymized data may be obtained from the corresponding author upon justified request.
