# Supplementary figures and images for: Digitally quantified area of residual tumor after neoadjuvant chemotherapy in HER2-positive breast cancer
Source: Breast Cancer. 2025 Apr 2;32(4):716–27. doi: 10.1007/s12282-025-01694-7 (PMC12174267; doi:10.1007/s12282-025-01694-7)

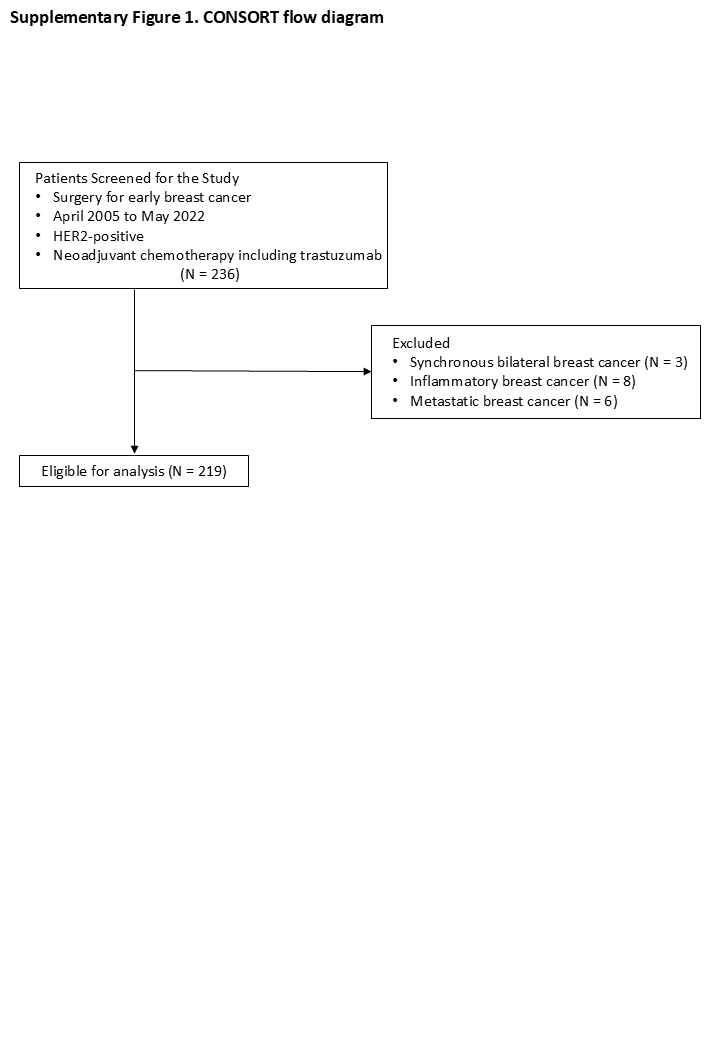

Supplement: Supplementary file 1 — Supplementary file1 (TIF 35 KB) [file 12282_2025_1694_MOESM1_ESM.tif]

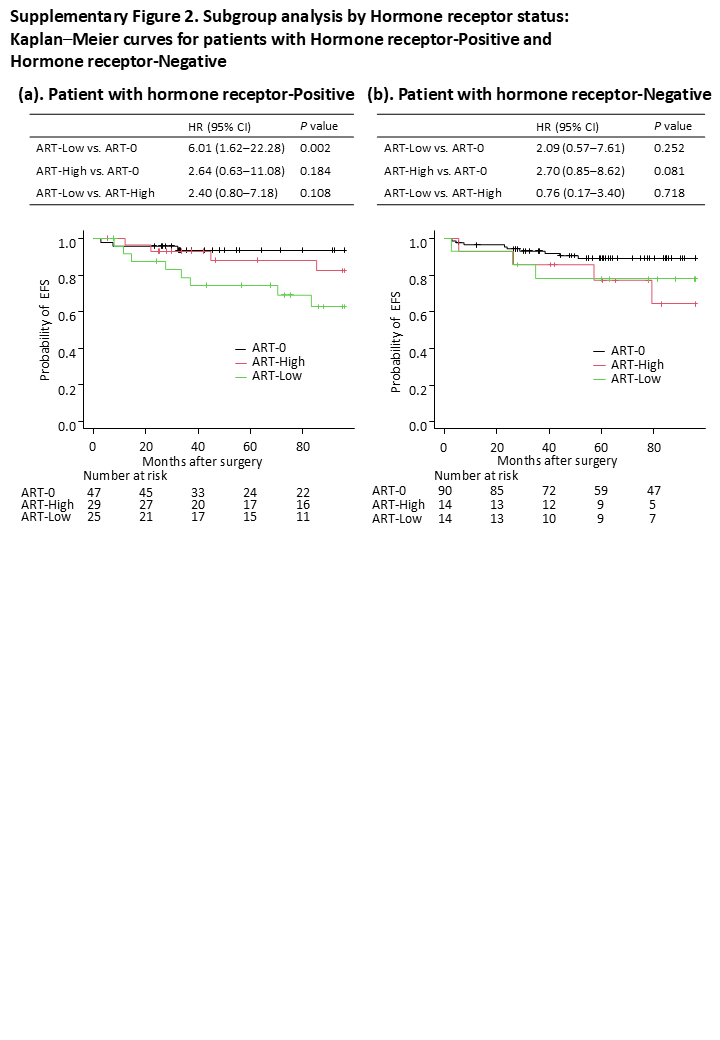

Supplement: Supplementary file 2 — Supplementary file2 (TIF 60 KB) [file 12282_2025_1694_MOESM2_ESM.tif]

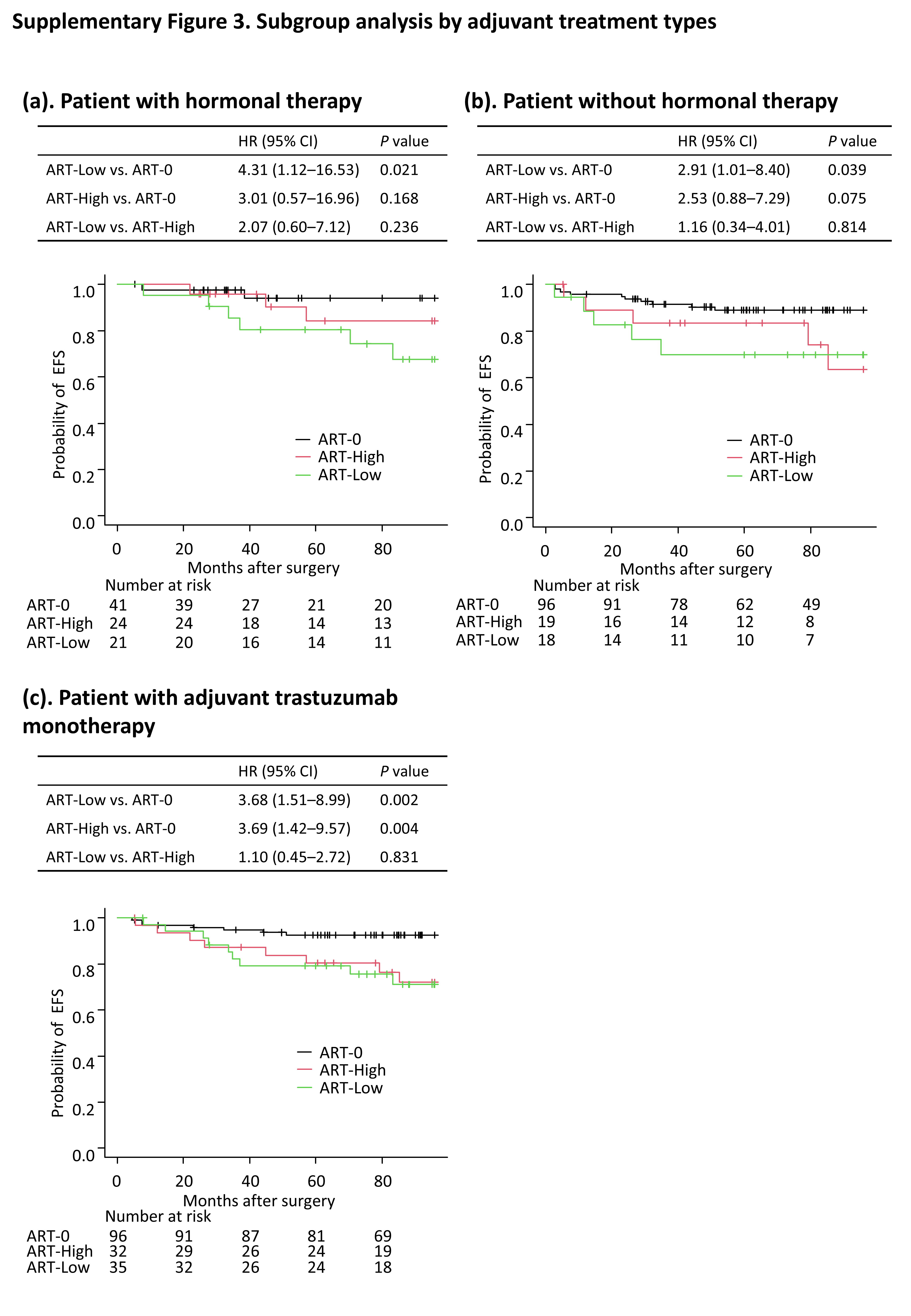

Supplement: Supplementary file 3 — Supplementary file3 (TIF 864 KB) [file 12282_2025_1694_MOESM3_ESM.tif]

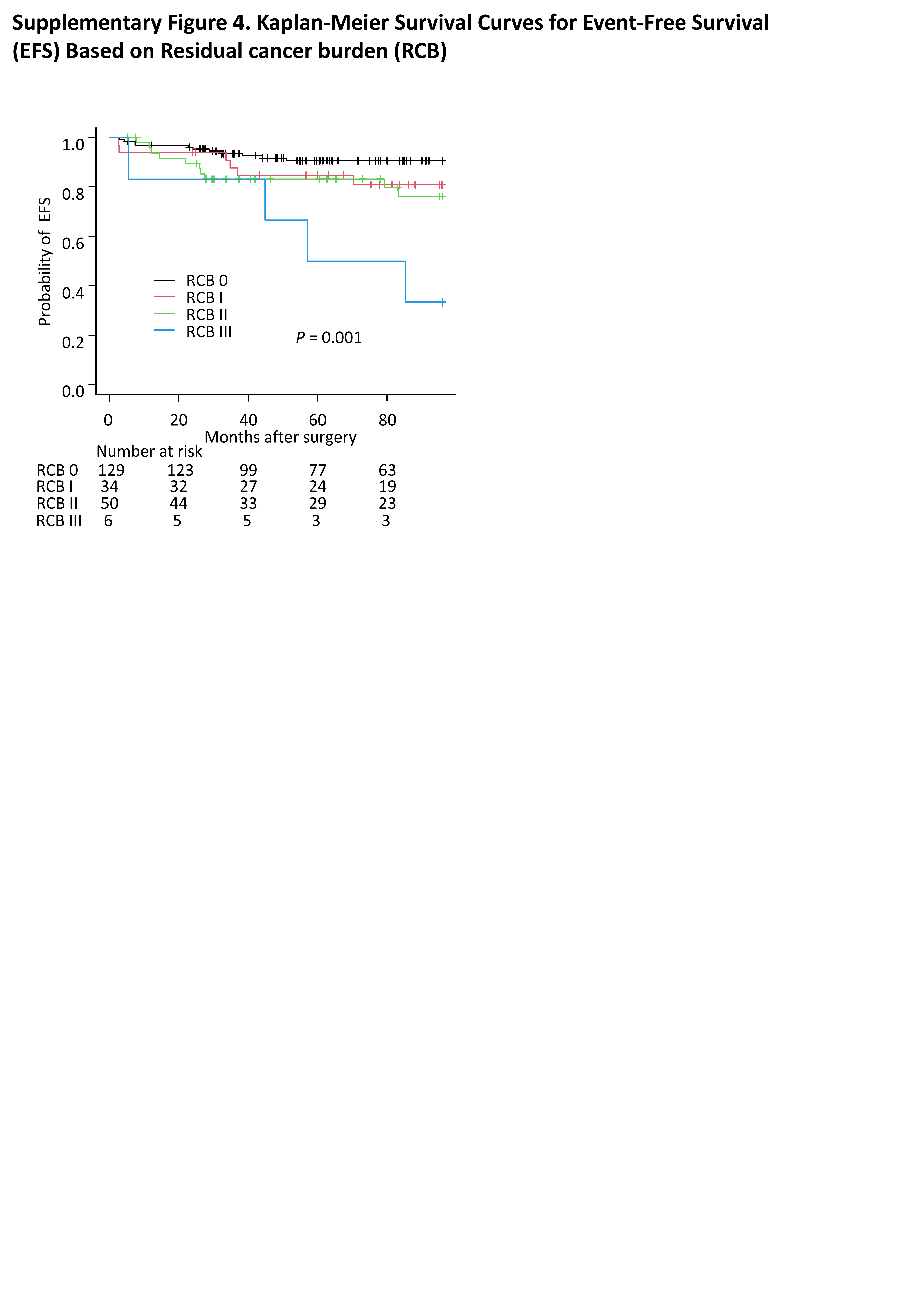

Supplement: Supplementary file 4 — Supplementary file4 (TIF 383 KB) [file 12282_2025_1694_MOESM4_ESM.tif]

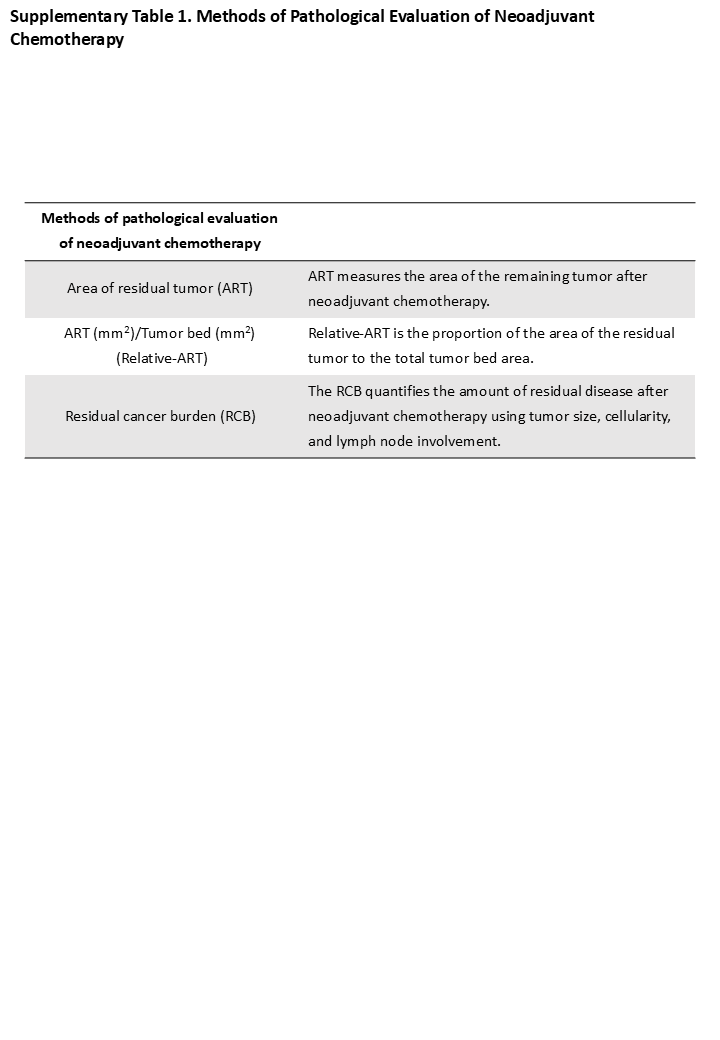

Supplement: Supplementary file 5 — Supplementary file5 (TIF 43 KB) [file 12282_2025_1694_MOESM5_ESM.tif]
